# Study on Data Partition for Delimitation of Masses in Mammography

**DOI:** 10.3390/jimaging7090174

**Published:** 2021-09-02

**Authors:** Luís Viegas, Inês Domingues, Mateus Mendes

**Affiliations:** 1 Polytechnic of Coimbra—ISEC, Rua Pedro Nunes, Quinta da Nora, 3030-199 Coimbra, Portugal; a21250789@isec.pt; 2 Medical Physics, Radiobiology and Radiation Protection Group, IPO Porto Research Centre (CI-IPOP), 4200-072 Porto, Portugal; inesdomingues@gmail.com; 3 ISR (Instituto de Sistemas e Robótica), Departamento de Engenharia Electrotécnica e de Computadores da UC, University of Coimbra, 3004-531 Coimbra, Portugal

**Keywords:** mammography, computer-aided detection, breast mass, mass detection, mass segmentation, Mask R-CNN, dataset partition

## Abstract

Mammography is the primary medical imaging method used for routine screening and early detection of breast cancer in women. However, the process of manually inspecting, detecting, and delimiting the tumoral massess in 2D images is a very time-consuming task, subject to human errors due to fatigue. Therefore, integrated computer-aided detection systems have been proposed, based on modern computer vision and machine learning methods. In the present work, mammogram images from the publicly available Inbreast dataset are first converted to pseudo-color and then used to train and test a Mask R-CNN deep neural network. The most common approach is to start with a dataset and split the images into train and test set randomly. However, since there are often two or more images of the same case in the dataset, the way the dataset is split may have an impact on the results. Our experiments show that random partition of the data can produce unreliable training, so the dataset must be split using case-wise partition for more stable results. In experimental results, the method achieves an average true positive rate of 0.936 with 0.063 standard deviation using random partition and 0.908 with 0.002 standard deviation using case-wise partition, showing that case-wise partition must be used for more reliable results.

## 1. Introduction

In 2020, there were 2.3 million new cases of brest cancer in the world [1]. That makes it the most common malignant tumor affecting women, accounting for a total of 11.7% of all cancer cases diagnosed. It is also the fifth leading cause of cancer mortality, with 685,000 deaths worldwide [1]. Among women, breast cancer is responsible for 1 in 4 cancer cases and 1 in 6 cancer-related deaths [1].

Despite these worrying figures, mortality from breast cancer is relatively low. In general, the disease has a good prognosis if the tumours are diagnosed in the early stages. About 90% of women with breast cancer are well five years after the original diagnosis [2]. However, due to the high incidence, this illness ranks first among all causes of cancer-related deaths in the female population. Mortality due to breast cancer has been decreasing continuously and consistently for several years. Early screening, that allows for the diagnosis of carcinomas at increasingly earlier stages, is one of the most important factors for the success of treatment and consequent reduction of mortality [3].

The present paper describes a method to detect and segment breast masses, based on a popular deep learning model known as Mask R-CNN. This model has been used before, with good results, by researchers such as Min et al. [4]. However, the focus of the present paper is a comparison to determine the importance of splitting the dataset properly, in order to avoid overfitting of the data. Experiments were performed splitting the images, to create the test set, randomly and by case. While this seems to be a small detail, in data preparation, it may have a significant impact on the results. The dataset used is the publicly available INbreast [5]. Experiments show that the method has competitive results compared to state-of-the-art methods. Additionally, division of the dataset by case instead of by image leads to more stable training procedures.

The paper is organized as follows: Section 2 explains in more detail what a mammogram image is and how computer aided detection can facilitate the diagnosis process. Section 3 presents a short survey of the state of the art related to detection and segmentation of masses from mammograms using deep learning. Section 4 describes methods to detect and segment tumoral masses. Section 5 contains a summary of the experiments and the results. Section 6 gives a brief discussion with comparison of results. Section 7 draws conclusions and highlights possible future research directions.

## 2. Mammography Images

Mammography has long been considered the most effective diagnostic imaging test for the early detection of breast cancer. The exam is simple and non-invasive. It must be performed routinely, in asymptomatic women (screening), or for diagnosis, being a fundamental tool in the detection of lesions in early stages, allowing a favorable prognosis and an increase in the success rate of treatments [6].

The imaging technique most used in the screening and diagnosis of breast cancer is X-ray mammography. It is a fast, low-cost technique with high spatial resolution. The basic views performed in a mammography exam are the Craniocaudal view (CC) and the Mediolateral Oblique view (MLO). Both are performed for each breast, up to a total of four images per patient. The main signs of breast cancer are the masses and clusters of microcalcifications, so the analysis of a mammographic image begins with the search for these types of formations.

There are different types of breast abnormalities. The abnormalities that can be seen in mammograms include masses, calcifications, asymmetry, or breast distortion. However, the breast masses, which are areas of thicker tissue that show in the mammography, are the most important sign of the illness. The analysis of mammogram images is a difficult task, even for trained radiologists. The main challenges are due to the different breast patterns, variations of color and shape of the tumoral masses, their possible locations, and different sizes possible. This variability often makes the abnormalities difficult to detect, segment, and classify.

The huge number of mammograms that can be generated and need to be analyzed during breast cancer screening programs require a significant workload, which often leads to fatigue and consequently errors of the radiologists that have to process and analyze hundreds or thousands of medical images over several days in a row. Therefore, Computer-Aided Detection (CAD) systems have been proposed, with the aim of assisting technicians and radiologists in the task, facilitating the process and contributing to lowering the probability of generating false negatives and false positives. CAD systems are used as a second opinion in the interpretation of mammograms, by the radiologists, contributing for more confidence in the diagnosis. However, such CAD systems need to operate at high levels of precision and accuracy. They must be robust, both to false positives and false negatives. A false positive can lead to unnecessary further testing, while a false negative can lead to further complications which might have been avoided.

The tumoral masses are volumes of abnormal density. Mammogram images are only an incomplete description of the 3D structure of the mass. The masses show in 2D mammography images with a high variability of shapes, sizes and locations. Most of the times they are difficult to distinguish from the background, even for experienced technicians. Existing CAD systems and modern detection and segmentation models have shown promising results, but the problem is still subject to heavy research. Training machine learning algorithms is also a challenge per se, for there are not many large datasets, containing Full Field Digital Mammograms (FFDM), annotated by experts and available for general use. This poses additional difficulties for developing modern CAD systems.

Recent developments in methods based on Deep Learning (DL) can contribute to develop robust solutions to undertake these problems. Particularly, the methods that use Convolutional Neural Networks (CNNs) to automatically learn a relevant hierarchy of features directly from inputting images. The topic has been subject to heavy research and there have been important developments. However, most developments are just in the specific area of detection, where the result is a bounding box [7], or in the specific area of region segmentation, to tell the region of interest from the background [8,9]. Nonetheless, there are also a number of important developments proposing a completely integrated system, able to detect and segment tumoral masses in the pipeline with minimal human intervention. The most common approaches still deal with two-dimensional images. Three-dimensional approaches have already been studied [10,11,12], and even stereoscopic approaches [13]. However, the state-of-the-art CAD systems are mostly based on 2D methods and trained on datasets consisting of 2D images. This makes the methods of pre-processing the images and partitioning the datasets a very important and still open issue.

## 3. Related Work

Tumor mass detection and segmentation in mammogram images have been subject to heavy research in recent years. One of the latest techniques to be applied is DL machine models, namely CNNs. CNNs have been applied in different medical image analysis with success. The review focuses on research papers that use the publicly available database INbreast, or other databases, for training and testing, having the focus on implementation of CNNs to address the issues of detection and/or segmentation of breast masses in mammograms.

### 3.1. Detection of Tumoral Masses

Many modern object detection models have achieved good performance in object detection and segmentation tasks. Nonetheless, those tasks still remain a challenge when detecting breast tumor masses in medical images, due to the low signal-to-noise ratio and the variability of size and shape of masses.

Dhungel et al. [14] presented an architecture that contains a cascade of DL and Random Forest (RF) classifiers for breast mass detection. Particularly, the system comprises a cascade of multi-scale Deep Belief Network (m-DBN) and a Gaussian Mixture Model (GMM) to provide mass candidates, followed by cascades of Region-based Convolutional Neural Network (R-CNNs) and RF to reduce false positives.

Wichakam et al. [15] proposed a combination between CNNs for feature extraction and Support Vector Machines (SVM) as the classifiers to detect a mass in mammograms. Choukroun et al. [16] presented a patch based CNN for detection and classification of tumor masses where the mammogram images are tagged only on a global level, without local annotations. The method classifies mammograms by detecting discriminative local information from the patches, through a deep CNN. The local information is then used to localize the tumoral masses.

### 3.2. Segmentation of Tumoral Masses

A fundamental stage in typical CAD systems is the segmentation of masses. Most popular segmentation approaches are based on pre-delimited Regions Of Interest (ROI) of the images.

Dhungel et al. [17] proposed the use of structured learning and deep networks to segment mammograms—specifically, using a Structured Support Vector Machine (SSVM) with a DBN as a potential function. In a first stage, the masses are manually extracted; then, a DBN is used to detect the candidates and a Gaussian Mixture Model classifier performed the segmentation step.

In [18,19], two types of structured prediction models are used, combined with DL based models as potential functions, for the segmentation of masses. Specifically, SSVM and Conditional Random Field (CRF) models were combined with CNNs and DBNs. The CRF model uses Tree Re-Weighted Belief Propagation (TRW) for label inference, and learning with truncated fitting. The SSVM model uses graph cuts for inference and cutting plane for training.

However, these methods [17,18,19] have some limitations due to their dependence on prior knowledge of the mass contour. Zhu et al. [20] proposed an end-to-end trained adversarial network to perform mass segmentation. The network integrates a Fully Convolutional Network (FCN), followed by a CRF to perform structured learning.

Zhang et al. [21] proposed a framework for mammogram segmentation and classification, integrating the two tasks into one model by using a Deep Supervision scheme U-Net model with residual connections.

Liang et al. [22] proposed a Conditional Generative Adversarial Network (CGAN) for segmentation of the tumoral masses in a very small dataset using only images with masses. The CGAN consists of two networks, the Mask-Generator and the Discriminator. The Mask-Generator network uses a modified U-Net, where the feature channels between low level feature layers are discarded, and the ones between high level feature layers are preserved. For the Discriminator network, a convolutional PatchGAN classifier is used. As a condition to achieve CGANs, an image sample with its ground truth is added into the Mask-Generator.

### 3.3. Detection and Segmentation of Masses

The approaches described above focus either on detection or on segmentation of the masses. However, there are also approaches that address both problems in a pipeline system. Pipeline techniques have recently received increasing attention in machine learning. A pipeline is created, so that successive transformations are applied on the data, the last being either a model training or prediction operation. The pipeline model is regarded as a block, connecting each task in the sequence to the successor and delivering the result at the end [23].

Sawyer Lee et al. [24] compare the performance of segmentation-free and segmentation-based machine learning methods applied to detection of breast masses. Rundo et al. [25] use genetic algorithms in order to improve the performance of segmentation methods in medical magnetic resonance images. Tripathy et al. [26] perform segmentation using a threshold method on mammogram images, after enhancing contrast using the CLAHE algorithm.

Some systems that integrate both detection and segmentation stage still require manual rejection of false positives before the segmentation stage, as happens in [27,28]. Dhungel et al. [27], presented a two-stage pipeline system for mass detection and segmentation. Specifically, they adopted a cascade of m-DBNs and GMM classifier to provide mass candidates. The mass candidates are then delivered to cascades of deep neural nets and random forest classifiers, for refinement of the detection results. Afterwards, segmentation is performed through a deep structured learning CRF model followed by a contour detection model.

Al-antari et al. [28] presented a serial pipeline system designed for detection, segmentation, and classification, also based on DL models. A YOLO CNN detector is implemented for mass detection. The results of the YOLO detector are then fed to an FCN to perform segmentation. The result is then fed to a basic deep CNN for classification of the mass as benign or malign.

In [29], the authors address detection, segmentation, and classification in a multi-task CNN model enabled by cross-view feature transferring. With an architecture built upon Mask R-CNN, the model enables feature transfer from the segmentation to the classification task to improve the classification accuracy.

Min et al. [4] presented a method for sequential mass detection and segmentation using pseudo-color mammogram images as inputs to a Mask R-CNN DL framework. During the training phase, the pseudo-color mammograms are used to enhance contrast of the lesions, compared to the background. That boosts the signal-to-noise ratio and contributes to improving the performance of the model in both tasks. The model comprises a Faster R-CNN object detector and an FCN for mask prediction. The method used for the experiments performed in the present work was based on the same framework. However, Min uses 5-fold cross validation, and this is not used in the present work.

## 4. Materials and Methods

The experiments were performed using an implementation of a Mask R-CNN to detect and segment tumoral masses in the INbreast dataset.

### 4.1. Database

The dataset used in the present study is obtained from INbreast, a publicly available full-field digital mammographic database with precise ground truth annotations [5]. The resolution of each image is 2560×3328 or 3328×4084 pixels, and they are in Digital Imaging and Communications in Medicine (DICOM) format. The confidential information was removed from the DICOM file but a randomly generated patient identification keeps the correspondence between images of the same patient. The database includes examples of normal mammograms, mammograms with masses, calcifications, architectural distortions, asymmetries, and images with multiple findings. For each breast, both CC and MLO views were provided. Among the 410 mammograms from 115 cases in INbreast, 107 contain one or more masses. There is a total of 116 benign or malignant masses. The average mass size is 479 mm^2^. The smallest mass has an area of 15 mm^2^, and the biggest one has an area of 3689 mm^2^.

The dataset is very small for training modern deep learning models, which require a large number of samples for proper training. However, large datasets are rare because of the difficulty in obtaining good quality medical images. Medical images require highly qualified people to provide the ground truth. There are also many privacy concerns because of the sensitive information they carry. Therefore, such images are rare and very important. Sometimes, the datasets are also imbalanced, with just a small number of samples showing a particular but important condition. Bria et al. [30] address the problem of class imbalance in medical images. A common technique is to use data augmentation, adding copies of some images with a transformation such as mirroring or rotation [31]. The present approach applies data augmentation through a random transformation, as described in Section 4.3.

### 4.2. Data Pre-Processing

One important step to start image processing is to tell the region of interest from the background. This can be done based on threshold methods [32]. Militello et al. use a different approach, based on quartile information [33], to distinguish epicardial adipose tissue from the background in medical cardiac CT scans. In the present work, the same procedure as in [4] was adopted. To prepare the images, the breast region is extracted using a threshold value to crop away the redundant background area. Specifically, and since the intensity of the background pixels of the INbreast mammograms is zero, the region where the pixels have a non-zero intensity value is extracted as the breast region [4,34]. The mammogram image is then resized to one fourth of the original image size. Afterwards, it is normalized to 16-bit. The normalized image is finally padded into a square matrix.

After cropping and normalization, the mammogram is converted to pseudo-color mammogram (PCM), in order to enhance the areas of thicker masses. The gray images were also changed to colour RGB images, which have the ability to convey colour information. In this way, the red, green and blue channels are filled respectively with the grayscale mammogram (GM), and two images generated by the Multi-scale Morphological Sifting (MMS) algorithm [4]. The images generated by MMS and the GM are linearly scaled to 8-bit. Therefore, a PCM RGB image comprises a GM in the first (R) channel, the output image of the MMS transform scale 1 into the second (G) channel and the MMS transform scale 2 in the third (B) channel.

The MMS makes use of morphological filters with oriented linear structuring elements to extract lesion-like patterns. The MMS can enhance lesion-like patterns within a specified size range. To deal with the size variation of breast masses, the sifting process is applied in two scales.

**Figure 1:**
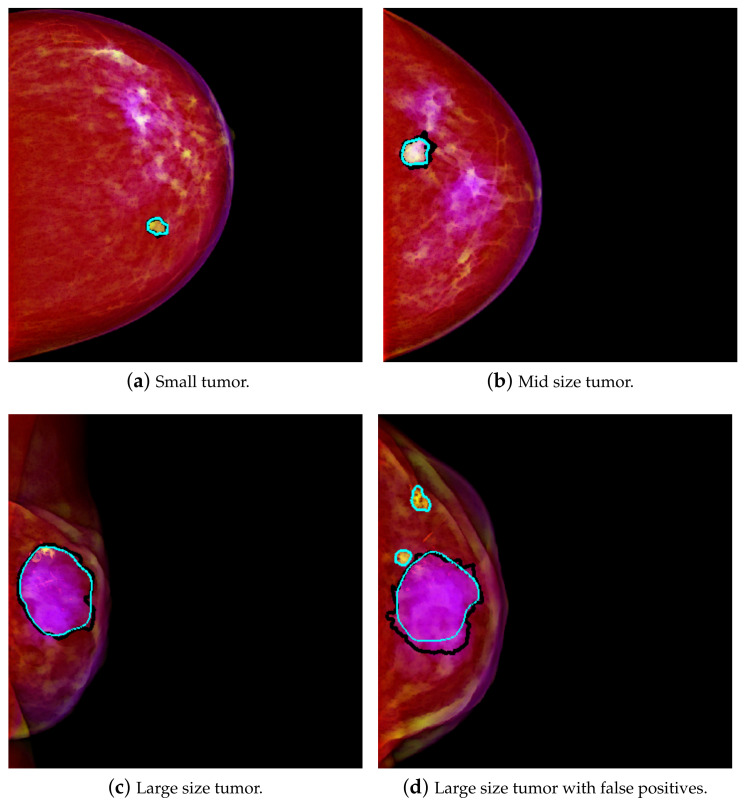
Some visual results of automatic detection and segmentation of breast masses. Black and cyan lines respectively stand for ground truth of the masses and segmentation of the detected regions.

The result is that a relatively smaller mass in the size range of scale 1 will have higher intensity in the second channel. Therefore, this is interpreted as a higher amount of green, and it tends to yellow on the PCM image. Figure 1a shows an example of a yellowish mass. A relatively larger size mass will have higher components in the range of scale 2, and therefore that will be interpreted as more of the blue component. The result is that it will tend to purple on the PCM image. This result is exemplified in Figure 1c. This transformation enhances the masses, which are then easier to differentiate from the background. As in [4], better results were achieved using PCM rather than using GM, so PCM was used for this work.

### 4.3. The Mask R-CNN Model

The present work applied transfer learning technique. Transfer learning is a common machine learning procedure where a pre-trained model is used as the basis to create a new model. In the present work, a pre-trained Mask R-CNN model was used, in order to speed up the training process. The dataset used is limited in size, thus starting with a pre-trained model not only speeds up the training process but also increases the chances of success. The Mask R-CNN is a framework that allows sequential mass detection and segmentation in mammograms. It integrates a Faster R-CNN object detector with an FCN for mask prediction. The Faster R-CNN utilizes the Region Proposal Network (RPN) to generate ROI candidates and then, for each candidate, performs classification and bounding-box regression. The FCN performs segmentation on the ROI candidates, generating the masks. During training, a multitasking loss function given by Equation (1) was used:

(1) $$L=L_{cls}+L_{bbox}+L_{msk}$$

where $L_{cls}$ is the classification loss, $L_{bbox}$ is the bounding box regression loss, and $L_{msk}$ is the mask loss, defined as the binary cross-entropy loss [35].

To make use of the transfer learning technique, the Mask R-CNN model training was initialized starting with the pre-trained “mask_rcnn_balloon” model. It consists of a network that was previously trained for a detection and binary classification problem of separation of balloons from the background [36].

A deep residual neural network, the ResNet101, was used as the model backbone. The images are resized into 1024×1024 pixels. To expand the number of images, data augmentation is implemented. Specifically, images are augmented by randomly selecting one of the available operations, namely, flipping up, down, left, right, and rotations in 90, 180 and 270 degrees. The network is then trained through 10 epochs, with a batch size of 1. The parameters settings mentioned above are the same as those utilized in [4]. For all the parameters which were not specified above, the default values in [36] were adopted.

For the experiments, we used Python 3.6 (available at http://www.python.org (accessed on 1 September 2021)) and ran on an Asus laptop with Intel(R) Core(TM) i7-7500U CPU @ 2.90 GHz, 16 GB RAM (Coimbra, Portugal).The generation of the pseudo-color image was implemented in MATLAB 2019b (available at https://www.mathworks.com/products/matlab.html (accessed on 1 September 2021)) using the same machine.

### 4.4. Evaluation Method

Experiments on the INbreast dataset were performed using all the 410 images available. Those 410 images must be split into at least the train and test set. Most of the experiments in the literature divide the data randomly, for example setting 70% for training, 15% for validation, and 15% for testing. However, as stated above, there are multiple images of the same patient and also of the same tumor. Therefore, some authors mention that data must be split case-wise to avoid contamination of the test and validation sets with images of patients or cases contained in the training set [16]. To the best of our knowledge, however, the impact of this possible contamination has not been tested before.

In the present work, different experiments were performed, with case-wise partition of the dataset and with random split partition. In all cases, the dataset was split into 280 images for training, 65 images for validation and 65 images for testing. Data augmentation doubles the number of images. In the case-wise partition, when performing the division, it was guaranteed that images of the same patient were in the same subset. The division is based on cases, ensuring that there were no case overlaps between the splits.

For the images with masses, segmentation masks are used as the ground truth, while, for the images without any masses, their ground truths are the black background.

For the evaluation of the performance of the method, the metrics used were Sensitivity (S) or True Positive Rate (TPR ) and False Positive Per Image (FPPI) for the mass detection task, and the Dice Similarity Index (Dice) for the mass segmentation task. The criteria for these metrics are defined as follows:

(2) $$\mathrm{TPR}=\frac{\mathrm{TP}}{\mathrm{TP}+\mathrm{FN}}$$

(3) $$\mathrm{FPPI}=\frac{\mathrm{FP}}{\mathrm{TP}+\mathrm{FP}}$$

(4) $$\mathrm{Dice}=\frac{2\times \mathrm{TP}}{2\times \mathrm{TP}+\mathrm{FP}+\mathrm{FN}}$$

where TP, FP, and FN represent the number of true positive, false positive and false negative detections, respectively. The mass is considered to be detected (TP) if the Intersection over Union (IoU) between the predicted bounding box and ground truth is greater than or equal to 0.2 [4].

## 5. Experiments and Results

Six experiments were performed. Three of them use random split partition of the images. The other three use case-wise partition. Mass detection and segmentation performance comparison between experiments are shown in Table 1. Experiments R1, R2 and R3 use random split of the images. Experiments C1, C2 and C3 use case-wise partition. The hyperparameters and other settings of the model were all the same, so that the results of the experiments could be compared.

More experiments could be performed for more confidence in the results. However, the results clearly show that case wise partition of the data seems to provide more stable results. In C1, C2, and C3, the TPR is very similar and the Dice only differed about 1%. Using randomly split data, however, the results for TPR varied between 0.875 and an overoptimistic 1.000 and the Dice varied between 0.857 and 0.885. In addition, R2 and R4 show a larger Dice variance than C1, C2 and C3.

The results show that using Mask R-CNN with PCMs, with case-wise dataset partition, achieves an average TPR of 0.909 at 0.77 FPPI and a Dice of 0.89 with some confidence on the results as shown in Table 1. The average TPR is 0.936 @ table. 1.30, with a standard deviation of 0.063 @ 0.19 using a random split of the samples. For case wise partition, the average is a bit lower, but the standard deviation is also lower: the average TPR is 0.908 @ 1.14 and the standard deviation is 0.002 @ 0.32. Thus, there is much less variation in the results obtained using case wise partition. As for Dice, using random split, the average Dice is 0.872 ± 0.086, with a standard deviation of 0.014 ± 0.038. The average Dice for mass segmentation using case wise partition is 0.889 ± 0.049, with a standard deviation of 0.009 ± 0.012. Therefore, in the case wise experiments, the standard deviation is always considerably lower than in the random split partition. Some visual results of detection and segmentation of breast masses are shown in Figure 1.

## 6. Discussion

Most medical image analysis applications require object detection, segmentation and classification. Modern DL models contribute to automation of all the tasks in a pipeline. Therefore, they are a useful technical solution to address the different tasks in a row.

The Mask R-CNN integrates mass detection and segmentation stages in one pipeline. Since a very small data set was used and training was initialized with pre-trained weights, there was no need to train for too long.

A public available dataset, INbreast, was used for evaluating the method. For quantitative analysis, three evaluation metrics, TPR or Recall, FPPI and Dice were utilized.

Case-wise partition was performed on dataset division to prevent images of the same case from appearing in more than one subset. Otherwise, contamination of the validation set and test set with images of the same patient could impact the results. This division by case seemed to have a small positive impact on the results obtained in the test set, compared to the results obtained when random split was used.

The global performance comparison between this method and several others methods are shown in Table 2. As the table shows, the results are competitive with the best published in the literature for the same dataset, and slightly better than other methods that perform detection and segmentation. Using case-wise partition, the results are also stable.

From Table 2, it can be seen that the PCMs + Mask R-CNN model, when compared to single task models, achieves a higher detection performance to [14,16], and outperforms [17,21] in segmentation. In addition, the model underperforms to a certain degree compared to [18,19,20] in segmentation. The reason may be that, in these [18,19,20], the input training samples were manually detected ROI masses, and this helped to improve the performance of segmentation results.

In comparison to Liang et al. [22], the method underperformed in segmentation. One of the reasons may be that Liang et al. used a very small and imbalanced dataset, consisting of only images with tumoral masses. In comparison with models which tackle both detection and segmentation, the model outperformed [27] in both tasks, achieving a similar sensibility and a higher segmentation performance than [29], and underperformed [28] in segmentation. For the lower result in comparison to [28], the reason may be that, like as in [27], they manually excluded all the false positive detections before segmentation. On the other hand, the PCMs + Mask R-CNN model is a fully automatic model, which can operate without human input.

## 7. Conclusions

An integrated mammographic CAD system based on deep learning is described. It is capable of simultaneous detection and segmentation of the masses, from mammograms based on Mask R-CNN. It does not require human intervention to operate.

Experimental results show that the system achieves state-of-the-art competitive performance in detection and segmentation. The results obtained from our experiments show that data preparation may have a small impact on the performance of the system. Namely, case-wide partition seems to have a small positive impact on the performance, preventing the system from overfitting compared to when the dataset is randomly split.

Future work includes tests with other datasets, as well as a study of the application of the methodology to other similar problems, such as other types of tumors. The method can also be tested with other medical imaging types and modalities, such as MRI and PET.

## Figures and Tables

**Table 1:** Comparison of TPR and Dice metrics between experiments. Experiments R1, R2 and R3 use a random split of the images. Experiments C1, C2 and C3 use case-wise partition of the images.

Experiment	TPR @ FPPI	Dice
R1	0.875 @ 1.47	0.885 ± 0.044
R2	0.933 @ 1.35	0.857 ± 0.118
R3	1.000 @ 1.09	0.874 ± 0.097
Average	0.936 @ 1.30	0.872 ± 0.086
STD	0.063 @ 0.19	0.014 ± 0.038
C1	0.909 @ 0.77	0.891 ± 0.050
C2	0.909 @ 1.32	0.880 ± 0.061
C3	0.906 @ 1.33	0.897 ± 0.036
Average	0.908 @ 1.14	0.889 ± 0.049
STD	0.002 @ 0.32	0.009 ± 0.012

**Table 2:** Performance comparison between PCMs + Mask R-CNN and several other state-of-the-art methods. The PCMs + Mask R-CNN is marked in bold.

Method	Database	TPR @ FPPI	Dice
Dhungel et al. [14]	INbreast	0.87 ± 0.14 @ 0.8	n.a.
Wichakam et al. [15]	INbreast	n.a.	n.a.
Choukroun et al. [16]	INbreast	0.76 @ 0.48	n.a.
Dhungel et al. [17]	INbreast	n.a.	0.88
Dhungel et al. [18]	INbreast	n.a.	0.90 ± 0.06
Zhu et al. [20]	INbreast	n.a.	0.9097
Dhungel et al. [19]	INbreast	n.a.	0.90
Zhang et al. [21]	INbreast	n.a.	0.85
Liang et al. [22]	INbreast	n.a.	0.91
Dhungel et al. [27]	INbreast	0.90 ± 0.02 @ 1.3	0.85 ± 0.02
Al-antari et al. [28]	INbreast	n.a.	0.9269
Gao et al. [29]	INbreast	0.91 ± 0.05 @ 1.5	0.76 ± 0.03
Min et al. [4]	INbreast	0.90 ± 0.05 @ 0.9	0.88 ± 0.10
**PCMs + Mask R-CNN**	**INbreast**	**0.909 @ 0.769**	**0.891 ± 0.05**

## Data Availability

Not applicable.

## Abbreviations

The following abbreviations are used in this manuscript:

CAD	Computer-Aided Detection system
CC	Craniocaudal
CGAN	Conditional Generative Adversarial Network
CNN	Convolution Neural Network
CRF	Conditional Random Fields
DBN	Deep Belief Network
DL	Deep Learning
DICOM	Digital Imaging and Communications in Medicine
GM	Grayscale Mammogram
GMM	Gaussian Mixture Model
Faster R-CNN	Faster Region based Convolutional Neural Network
FCN	Fully Convolutional Network
FFDM	Full Field Digital Mammogram
FPPI	False Positive Per Image
FrCN	Full Resolution Convolutional Network
IoU	Intersection over Union
m-DBN	multi-scale Deep Belief Network
MG	Mammogram
MLO	Mediolateral Oblique
MMS	Multi-scale Morphological Sifting
MRI	Magnetic Resonance Imaging
MTL	Multi-Task Learning
PCM	Pseudo-Color Mammogram
PET	Positron Emission Tomography
R-CNN	Region based Convolutional Neural Network
RF	Random Forest
RGB	Red, Green, Blue color model
RPN	Region Proposal Network
ROI	Region Of Interest
SSVM	Structured Support Vector Machine
SVM	Support Vector Machine
TPR	True Positive Rate
TRW	Tree Re-Weighted Belief Propagation
YOLO	You-Only-Look-Once
